# An Evaluation of the DNA-Protective Effects of Extracts from *Menyanthes trifoliata* L. Plants Derived from *In Vitro* Culture Associated with Redox Balance and Other Biological Activities

**DOI:** 10.1155/2019/9165784

**Published:** 2019-10-16

**Authors:** Tomasz Kowalczyk, Przemysław Sitarek, Ewa Skała, Patricia Rijo, Joana M. Andrade, Ewelina Synowiec, Janusz Szemraj, Urszula Krajewska, Tomasz Śliwiński

**Affiliations:** ^1^Department of Molecular Biotechnology and Genetics, University of Lodz, Banacha 12/16, 90-237 Lodz, Poland; ^2^Department of Biology and Pharmaceutical Botany, Medical University of Lodz, Muszynskiego 1, 90-151 Lodz, Poland; ^3^Center for Research in Biosciences and Health Technologies (CBIOS), Universidade Lusófona de Humanidades e Tecnologias, 1749-024 Lisbon, Portugal; ^4^Instituto de Investigação do Medicamento (iMed.ULisboa), Faculdade de Farmácia, Universidade de Lisboa, 1649-003 Lisbon, Portugal; ^5^Laboratory of Medical Genetics, Faculty of Biology and Environmental Protection, University of Lodz, Pomorska 141/143, 90-236 Lodz, Poland; ^6^Department of Medical Biochemistry, Medical University of Lodz, Mazowiecka 6/8, 92-215 Lodz, Poland; ^7^Department of Pharmaceutical Biochemistry, Molecular Biology Laboratory, Medical University of Lodz, Muszynskiego 1, 90-151 Lodz, Poland

## Abstract

*Menyanthes trifoliata* L. is a valuable medical plant found in Europe, North America, and Asia, which grows on peat bogs and swamps. It has long been used in folk medicine as a remedy for various ailments. This is the first report to demonstrate the protective antioxidant and anti-inflammatory properties of aqueous methanolic extracts derived from the aerial parts (MtAPV) and roots (MtRV) of *in vitro* grown plants on human umbilical vein endothelial cells (HUVECs). It describes the influence of the tested extracts on the expression of antioxidant (HO-1, NQO1, *NRF2*, *kEAP1*, and *GCLC*) and inflammation-related genes (*IL-1α*, *IL-1β*, *IL-6*, *TNF-α*, and *IFN-γ*) in cells stimulated with H_2_O_2_ or LPS, respectively. In addition, *M. trifoliata* extracts were found to moderately affect the growth of certain bacterial and fungal pathogens, with the strongest antibacterial effect found against *Pseudomonas aeruginosa* and *Enterococcus faecalis*. *M. trifoliata* extracts demonstrated protective effects against mitochondrial DNA (mtDNA) and nuclear DNA (nDNA) damage caused by ROS, decreasing the numbers of mtDNA lesions in the *ND1* and *ND2* genes and nDNA damage in the *TP53* and *HPRT1* genes and reducing cleavage in PARP1- and *γ*-H2A.X-positive cells. The root extract of *in vitro M. trifoliata* (MtRV) appears to have better anti-inflammatory, antioxidant, antimicrobial, and protective properties than the extract from the aerial part (MtAPV). These differences in biological properties may result from the higher content of selected phenolic compounds and betulinic acid in the MtRV than in the MtAPV extract.

## 1. Introduction

Health-promoting properties of plants have been used in the prevention and therapy of many human diseases for thousands of years. Currently, it is estimated that 300,000 plant species exist worldwide [1]; however, relatively few have confirmed therapeutic or protective properties. Fortunately, modern methods and equipment allow much faster and more accurate analyses of the resources hidden in plants. It is well known that they synthesize a large number of metabolites which play important roles, including defense against herbivores, other plants, or pathogens, and some can be successfully used in general health care [2–5]. One such group of extremely valuable plant secondary metabolites is the polyphenols, which allow the plant to respond to stress agents. When assimilated through the diet in fruits, vegetables, tea, or medicinal and culinary herbs, these natural compounds play a key role in the antioxidant protection of the human body and are believed to have a significant impact on reducing the risk of cancer or cardiovascular disease [6]. Therefore, there is a need to identity rich sources of these compounds in nature and study their biological properties.

*Menyanthes trifoliata* L., Menyanthaceae, offers a lot of promise. This medicinal plant, commonly known as Bogbean, occurs in the northern hemisphere, mainly in the circumpolar temperate zone of Europe, North America, and Asia [7]. In traditional and folk medicine, its leaves are used to treat lack of appetite, scurvy, fevers, and skin disorders. Extracts obtained from *in vitro* cultures of this plant have also been found to induce apoptosis in glioma cells [8]. Studies have examined the biological properties of its metabolites [9, 10], and their results indicate that *M. trifoliata* is a source of phenolics or flavonols [11], some of which, such as phenolic acids, scopoletin, rutin, or loganin, may have potential medical applications [12–14].

The aim of this work is to determine the antioxidant, anti-inflammatory, antimicrobial, and DNA-protective effects of the aerial part and root extracts from *Menyanthes trifoliata* plants grown in *in vitro* culture on Schenk and Hildebrandt (SH) medium together with various other biological properties.

## 2. Materials and Methods

### 2.1. Plant Material

**Figure 1:**
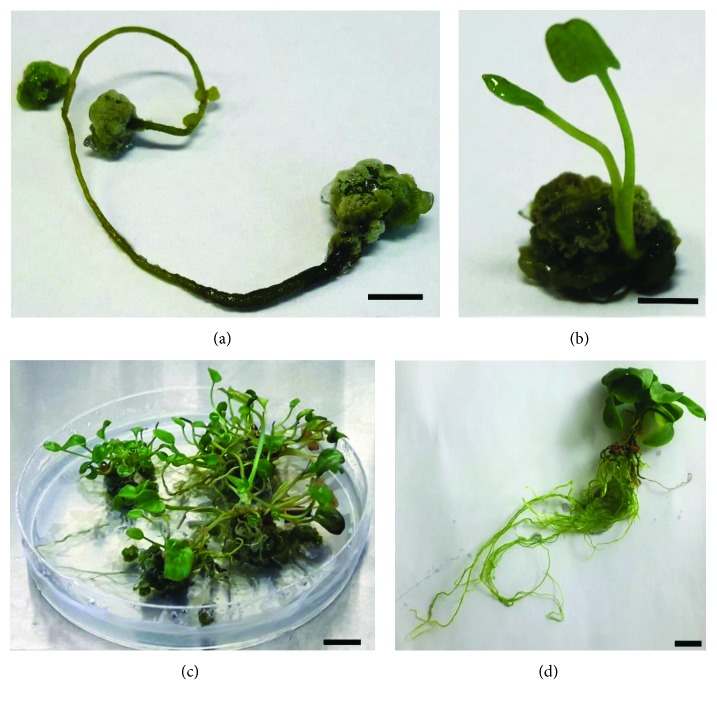
Subsequent stages of *Menyanthes trifoliata* regeneration. (a) Callus formation on the root after 20-day incubation on SH medium with 1.0 mg/L (6-BA) and 0.1 mg/L (NAA). (b) Regenerated shoot formed on SH medium with 4.0 mg/L 6-BA and 0.3 mg/L NAA. Fourth week of incubation. (c) Rooted young plants grown on SH medium supplemented with 0.3 mg/L 6-BA and 1.0 mg/L NAA before transfer to fresh solid SH medium. (d) Regenerated plant grown on solid SH medium without growth regulators after five weeks. Bar = 1 cm.

*In vitro* shoots of *M. trifoliata* were established from seeds as described previously [8]. In the present study, young leaves, roots, and stems were used for shoot regeneration. The explants were cultured on 0.8% agar-solidified Schenk and Hildebrandt (SH) medium supplemented with 1.0 mg/L 6-benzyladenine (6-BA) and 0.1 mg/L *α*-naphthaleneacetic acid (NAA) under the following conditions: 16/8-hour light/dark photoperiod; light intensity, 40 *μ*mol m^−2^ s^−1^; and temperature, 26°C. Light green calli were observed after 14 days on the surface of explants; these were then transferred to SH medium with 4.0 mg/L 6-BA and 0.3 mg/L NAA for shoot induction. After four weeks, the shoots obtained from calli were transferred to SH rooting medium supplemented with 0.3 mg/L 6-BA and 1.0 mg/L NAA. After two weeks, rooted young plants were transferred to solid SH medium without growth regulators and cultured under the conditions described above. In *M. trifoliata in vitro*, propagation steps are shown in Figures 1(a)–1(d).

### 2.2. *M. trifoliata* Aerial Part and Root Extract Preparation

The plant extracts from aerial parts and roots of *M. trifoliata* plants grown *in vitro* were prepared according to Sitarek et al. [15]. Briefly, 10 g of dry weight of aerial parts and roots was used for extraction. The plant material was extracted for 15 minutes with 80% (*v*/*v*) aqueous methanol (500 mL) at 35°C using an ultrasonic bath and then for 15 minutes with 300 mL of the same solvent. Finally, the extracts were filtered, evaporated under reduced pressure, lyophilized to dryness, and then kept dry in the dark until further use. The yields (*w*/*w*) of the extracts were 52.8% and 50.4% of initial plant dry weight for aerial parts (MtAPV) and roots (MtRV) of *M. trifoliata* plants, respectively.

### 2.3. Phytochemical Analysis of Extracts of *M. trifoliata* Aerial Parts and Roots

The phenolic compounds and betulinic acid were examined in MtAPV and MtRV extracts by HPLC (Dionex, Sunnyvale, USA) according to Kowalczyk et al. [8].

### 2.4. Cell Cultures

All experiments were performed on human umbilical vein endothelial cells (HUVECs). These cells were purchased from Gibco (Cascade Biologics®, catalog number C0035C) and cultured in Medium 200 (Gibco, catalog number M-200-500) supplemented with Low Serum G Growth Supplement Kit (LSGS Kit; Gibco, catalog number S003K) at 37°C and 5% CO_2_ in an incubator (Galaxy® 170 R-CO_2_ Incubator, New Brunswick Scientific) under a humidified atmosphere.

### 2.5. Cell Viability

The MTT assay was employed to measure the viability of HUVECs treated with different concentrations of LPS or H_2_O_2_ and the MtRV and MtAPV extracts of *M. trifoliata* plants grown *in vitro*. Briefly, cells were seeded at 1 × 10^4^ cells per well in 96-well culture plates and were left overnight before being treated for attachment. The cells were incubated for 24 hours with MtRV and MtAPV extracts over the concentration ranges 0-5 mg/mL. The inflammatory process was initiated with 1 *μ*g/mL LPS, and separately, the antioxidant response was stimulated with 50 *μ*M H_2_O_2_. In the next experiment, the cells were incubated with 1 mg/mL MtRV and MtAPV extracts or pretreated with LPS or H_2_O_2_ for one hour and then treated with plant extracts for 24 hours. After completing the incubation, the cells were washed once and incubated with 0.5 mg/mL of 3-(4,5-dimethylthiazol-2-yl)-2,5-diphenyl tetrazolium bromide (MTT) at 37°C for four hours. The MTT was then carefully removed and DMSO (100 *μ*L) added to each well. The mixture was vortexed at low speed for five minutes to fully dissolve the blue crystals. Absorbance was measured at 570 nm with a reference at 630 nm using a BioTek Synergy HT Microplate Reader (BioTek Instruments, Winooski, VT, USA). Independent experiments were repeated in triplicate. Cell viability was expressed as a percentage relative to the untreated (control) cells, which was defined as 100%.

### 2.6. Gene Expression

HUVECs were first incubated in the presence of LPS or H_2_O_2_ for one hour and then with 1 mg/mL MtRV and MtAPV extracts for 24 hours. Total RNA was then extracted using an ISOLATE II RNA Mini Kit, according to the manufacturer's instructions. cDNA was synthesized from total RNA using a High-Capacity cDNA Reverse Transcription Kit. A sample of 1 ng total RNA was used as a template in a total volume of 10 *μ*L, following the manufacturer's instructions. Next, gene expression was analyzed by TaqMan Probe-Based Real-Time PCR Assay. TaqMan probes (Life Technologies) were used to analyze 10 genes (*HO-1*, *NQO1, kAEP1*, *GCLC*, *IL-1α*, *IL-1β*, *IL-6*, *TNF-α*, and *IFN-γ*), and beta actin (*ACTB*) was included as the reference gene. qRT-PCR was performed using TaqMan® Real-Time PCR Master Mix (Life Technologies) and a CFX96™ Real-Time PCR Detection System (Bio-Rad Laboratories, Hercules, CA, USA) working on CFX Manager™ Software (version 3.1). The thermal cycling conditions were as follows: 10 minutes of polymerase activation at 95°C, followed by 40 cycles of 30 s denaturation at 95°C and 60 s annealing/extension at 60°C. Each sample was run in triplicate. The basal expression level was calculated using the *C*_t_ method [16].

### 2.7. Determination of Nuclear DNA (nDNA) Damage and Mitochondrial DNA (mtDNA) Damage

Total genomic DNA (nuclear and mitochondrial) was isolated from cells treated with 1 mg/mL MtRV and MtAPV extracts for 24 h and then treated with 50 *μ*M H_2_O_2_ for one hour using a QIAamp DNA Mini Kit (Qiagen, Valencia, CA, USA) according to the manufacturer's instructions. DNA concentrations were determined by spectrophotometric measurement of absorbance at 260 nm, and the purities were calculated by the A260/A280 ratio using a BioTek Synergy HT Microplate Reader (BioTek Instruments, Winooski, VT, USA). The semilong run quantitative RT-PCR (SLR-qRT-PCR) was used to assess mitochondrial DNA (mtDNA) and nuclear DNA (nDNA) damage, as described previously, with some modifications [17]. All conditions were described previously [18].

### 2.8. Cytoplasmic ROS and Mitochondrial ROS Detection

The cells were incubated with 1 mg/mL MtRV or MtAPV for 24 hours and then with 50 *μ*M H_2_O_2_ for one hour. DCFDA (molecular probe, Life Technologies) staining was used to detect cytoplasmic ROS. DCFDA was diluted to a 5 *μ*mol/L final concentration with Hanks' balanced salt solution (HBSS), which was incubated with the cells at 37°C for 30 minutes. A MitoSOX Red mitochondrial superoxide indicator was used to measure mitochondrial ROS production. The indicator was diluted to a 5 *μ*mol/L final concentration with HBSS, which was applied to incubate cells at 37°C for 30 minutes. The cells were washed three times with HBSS. The fluorescence emission was measured using a BioTek Synergy HT Microplate Reader. Each sample was run in triplicate.

### 2.9. Analysis of Cellular Phosphorylated H2A.X and Cleaved PARP Levels

HUVECs were plated in a six-well plate at a density of 2 × 10^5^ viable cells. The following day, the cells were treated with MtRV and MtAPV extracts (1 mg/mL) for 24 h and then incubated with 50 *μ*M H_2_O_2_ for one hour. After incubation, the cells were collected, and phosphorylated H2A.X- and cleaved PARP-positive cells were detected using the Apoptosis, DNA Damage and Cell Proliferation Kit (BD Pharmingen, 562253) according to the protocol given by the manufacturer. The cells were analyzed with a FACSCanto II cytometer (Becton Dickinson, San Jose, California, USA).

### 2.10. Antimicrobial Activity

The following strains were tested: *Staphylococcus aureus* (ATCC 25923), *Enterococcus faecalis* (ATCC29212), *Escherichia coli* (ATCC 25922), *Pseudomonas aeruginosa* (ATCC 27853), and the yeasts *Candida albicans* (ATCC 10231) and *Saccharomyces cerevisiae* (ATCC 2601). The growth conditions of all the tested microorganisms were described previously by Sitarek el al. [19]. MtRV and MtAPV extracts were used in this study. The minimum inhibitory concentration (MIC) and the Minimum Bactericidal/Fungicidal Concentration (MBC or MFC) of the tested extract were determined as detailed previously [19].

### 2.11. Data Analysis

The statistical analysis was performed using GraphPad Prism 5. All experimental values presented in this study were expressed as mean ± standard deviation (SD). The Shapiro-Wilk test to analyze the normality of the distribution of results was used. Next, the results were analyzed for equality of variance using Levene's test. Significant differences were identified using the ANOVA test for multiple comparisons, followed by the Dunnet *post hoc* test.

## 3. Results

### 3.1. Chemical Composition of Aerial Part (MtAPV) and Root Extracts (MtRV) of *M. trifoliata* Plants

The following phenolic compounds have previously been identified in MtRV and MtAPV extracts: chlorogenic acid (177 and 258 *μ*g/g dry weight, respectively), ellagic acid (518 and 451 *μ*g/g dry weight, receptively), sinapinic acid (146 and 71 *μ*g/g dry weight, respectively), syringic acid (114 *μ*g/g dry weight and not detected, respectively), rutin (256 and 153 *μ*g/g dry weight, respectively), and also pentacyclic triterpene-betulinic acid (5437 and 395 *μ*g/g dry weight, respectively) [8]. The main components of the plant extracts are presented in Table 1.

### 3.2. Effects of MtAPV and MtRV Extracts on Cell Viability

**Figure 2:**
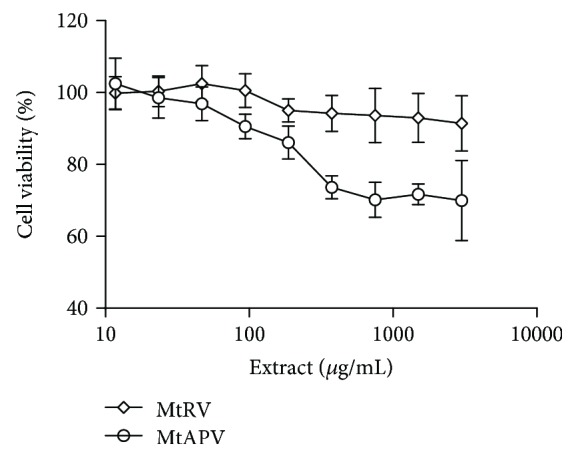
Cell viability after 24-hour treatment of MtRV and MtAPV extracts of *M. trifoliata* plants at concentrations of 0-5 mg/mL on HUVECs. Data are presented as mean ± SD.

Our results showed that the MtRV and MtAPV extracts of *M. trifoliata* plants did not present any cytotoxic effect on the human umbilical vein endothelial cells (HUVECs) in the tested concentrations (0-5 mg/mL) after 24-hour incubation (Figure 2).

**Figure 3:**
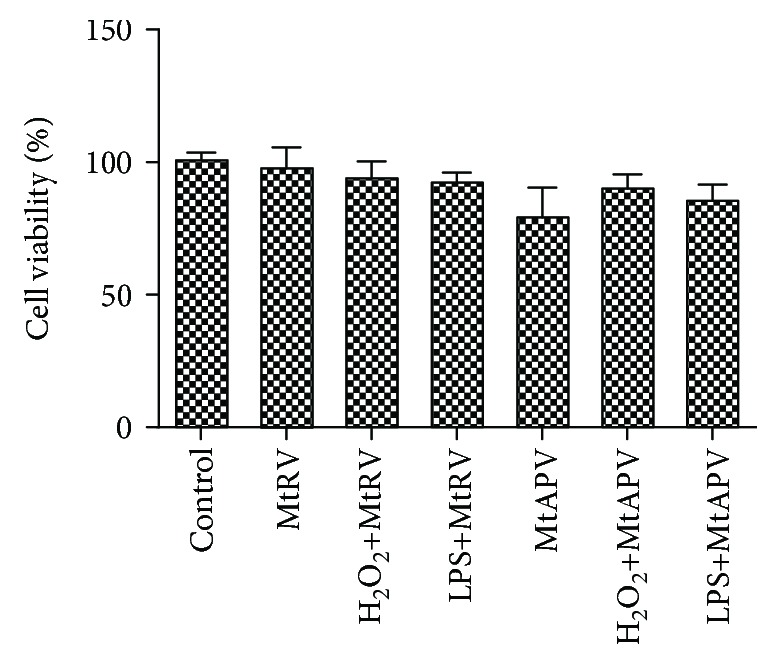
Cell viability after 24-hour treatment of HUVECs with 1 mg/mL of *M. trifoliata* MtRV and MtAPV extracts and pretreatment with LPS and H_2_O_2_. Data are presented as mean ± SD.

In further experiments, 1 *μ*g/mL LPS or 50 *μ*M H_2_O_2_ was used: these values did not exhibit 50% growth inhibition (IC_50_ value) of cells and were used for the induction of inflammation or antioxidant effect, respectively (data not shown). In addition, the effect on cell viability was determined for both extracts of *M. trifoliata* in combination with LPS or H_2_O_2_. It was found that the HUVECs demonstrated no significant difference in cell viability following treatment with MtRV or MtAPV extracts alone, MtRV or MtAPV in combination with H_2_O_2_, and also MtRV or MtAPV in combination with LPS (Figure 3).

### 3.3. Gene Expression

**Figure 4:**
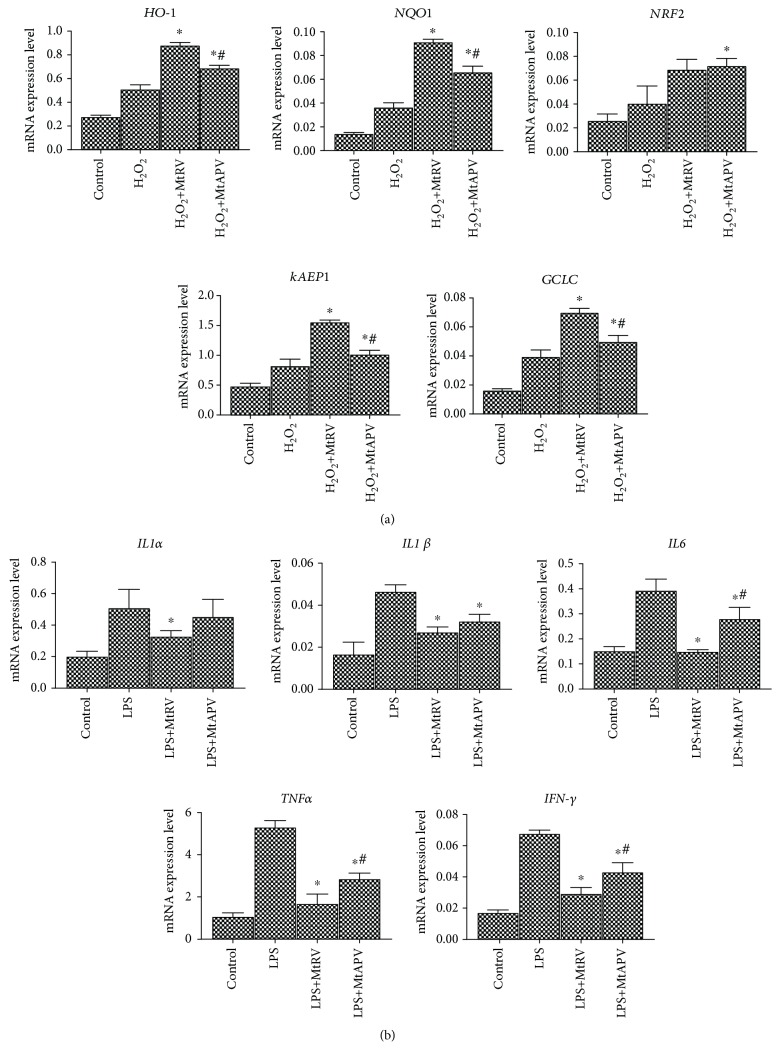
(a) mRNA expression of antioxidant genes after 24-hour treatment of MtRV and MtAPV extracts (1 mg/mL) of *M. trifoliata* plants and stimulation with 50 *μ*M H_2_O_2_. (b) mRNA expression of inflammatory genes after 24-hour treatment with MtRV and MtAPV extracts of *M. trifoliata* plants and 1 *μ*g/mL LPS. ^∗^For comparison control vs. MtAPV and MtRV extracts. ^#^For comparison MtAPV vs. MtRV extracts. *ACTB* served as the reference gene. Data are presented as mean ± SD (*n* = 3).

Quantitative real-time RT-PCR was used to measure mRNA levels of the antioxidant genes HO-1, NQO1, *NRF2*, *kAEP1*, and *GCLC* and inflammation genes *IL-1α*, *IL-1β*, *IL-6*, *TNF-α*, and *IFN-γ* after treatment with 50 *μ*M H_2_O_2_ or 1 *μ*g/mL LPS for one hour and after treatment with the MtRV or MtAPV extract of *M. trifoliata* for 24 hours. Our results demonstrated that treatment with both extracts at a concentration of 1 mg/mL was able to change the level of antioxidant genes in H_2_O_2_-stimulated HUVECs compared to the controls. Similar results were obtained for inflammatory genes. All the tested genes in the LPS-stimulated cells demonstrated changes in expression following treatment with either extract compared to controls, but better anti-inflammatory properties were observed for the MtRV extract. The results are shown in Figures 4(a) and 4(b).

### 3.4. Quantification of Nuclear DNA (nDNA) and Mitochondrial DNA (mtDNA) Damage

**Figure 5:**
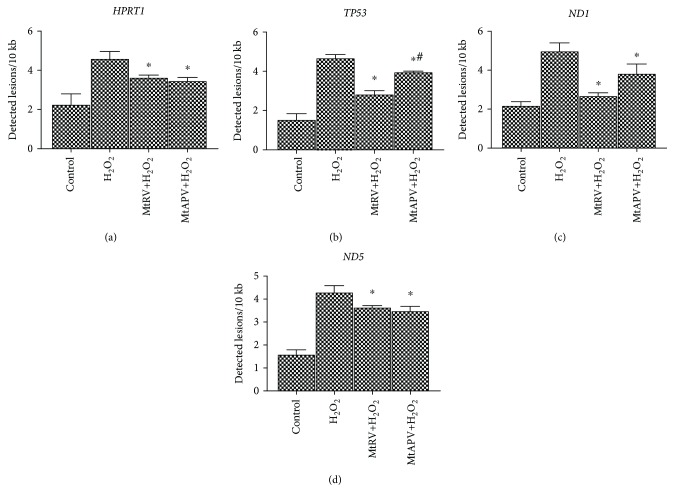
Quantification of nuclear DNA (nDNA) (a, b) and mitochondrial DNA (mtDNA) (c, d) lesion frequency per 10 kb DNA by SLR-qRT-PCR amplification of total DNA from human umbilical vein endothelial cells exposed to MtRV and MtAPV (1 mg/mL) extracts for 24 hours followed by 50 *μ*M H_2_O_2_ for one hour. ^∗^For comparison control vs. MtAPV and MtRV extracts. ^#^For comparison MtAPV vs. MtRV extracts. Data represent the mean ± SD of three replicates.

mtDNA and nDNA damage was examined by SLR-qRT-PCR amplification of DNA isolated from HUVECs exposed to MtRV or MtAPV extracts for 24 hours and then 50 *μ*M H_2_O_2_ for one hour. The cells exposed to 50 *μ*M H_2_O_2_ demonstrated a significant increase in lesion rate in the *ND1* and *ND5* regions: about five lesions per 10 kb DNA. Similarly, the same cells demonstrated an increase in lesion rate in the *HPRT1* and *TP53* regions of nDNA: about five lesions per 10 kb DNA were observed. In the cells, 24-hour treatment with MtRV or MtAPV extracts was found to bestow a protective effect against mtDNA and nDNA damage by decreasing the mtDNA lesion number in the *ND1* and *ND5* genes and nDNA damage in the *TP53* and *HPRT1* genes (Figures 5(a)–5(d)).

### 3.5. Cytoplasmic ROS and Mitochondrial Superoxide Generation Detection

**Figure 6:**
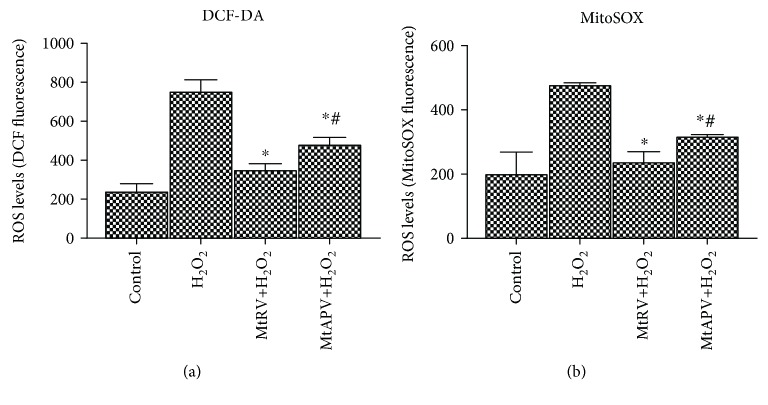
The production of ROS in human umbilical vein endothelial cells exposed to MtRV and MtAPV extracts of *M. trifoliata* (1 mg/mL) for 24 hours followed by 50 *μ*M H_2_O_2_ for one hour, measured using the probes: DCFDA (a) and MitoSOX (b). ^∗^For comparison control vs. MtAPV and MtRV extracts. ^#^For comparison MtAPV vs. MtRV extracts. Results represent means ± SD.

Cytoplasmic and mitochondrial ROS levels were measured after 24-hour treatment with MtRV and MtAPV (1 mg/mL) extracts and then one-hour treatment with 50 *μ*M H_2_O_2_. Following 24-hour treatment with either tested extract, decreased cytoplasmic and mitochondrial ROS levels were observed compared to the cells treated with only H_2_O_2_, indicating the presence of a protective effect (Figures 6(a) and 6(b)).

### 3.6. Assessment of PARP1- and *γ*-H2A.X-Positive Cell Levels by Flow Cytometry after Treatment with MtAPV and MtRV Extracts

**Figure 7:**
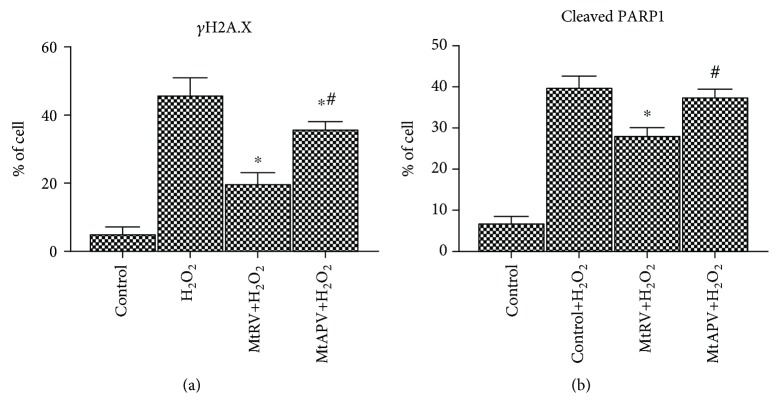
Diagrams presented percentage of H2A.X phosphorylation- (a) and cleaved PARP1- (b) positive human umbilical vein endothelial cells after 24-hour exposure to MtRV and MtAPV extracts followed by 50 *μ*M H_2_O_2_ for one hour. ^∗^For comparison control vs. MtAPV and MtRV extracts. ^#^For comparison MtAPV vs. MtRV extracts. The values represent mean ± SD of three independent experiments.

The levels of *γ*H2A.X- and cleaved poly (ADP-ribose) polymerase 1- (PARP1-) positive cells were evaluated to determine the protective effect of MtRV and MtAPV extracts on HUVECs induced by 50 *μ*M H_2_O_2_. It was found that the HUVECs treated with 50 *μ*M H_2_O_2_ generated a higher level of *γ*H2A.X- and cleaved PARP1-positive cells than untreated cells. In turn, 24-hour incubation of cells with MtRV and MtAPV extracts demonstrated a protective effect on cells following treatment with 50 *μ*M H_2_O_2_, reflecting a significantly lower percentage of *γ*H2A.X- and cleaved PARP1-positive cells. Although both extracts demonstrated a protective effect, better results were observed for the MtRV extract (Figures 7(a) and 7(b)).

### 3.7. Assessment of Antimicrobial Activity of MtAPV and MtRV Extracts

The antimicrobial activity of MtRV and MtAPV extracts against *Staphylococcus aureus*, *Pseudomonas aeruginosa*, *Escherichia coli*, *Enterococcus faecalis*, *Saccharomyces cerevisiae*, and *Candida albicans* was screened using MIC and MBC/MFC methods. Both the tested extracts showed moderate antimicrobial activity (Table 2) against various strains at a range of MIC values (150-925 *μ*g/mL) and for MBC/MFC (500-2500 *μ*g/mL). The MtRV extract showed better activity than the MtAPV extract against *P. aeruginosa* and *E. faecalis*, with a MIC value of 150 *μ*g/mL. The MtRV extract demonstrated also better antifungal activity than the MtAPV extract for *C. albicans* and *S. cerevisiae*, with MIC values of 625 *μ*g/mL and 725 *μ*g/mL, respectively, and with MFC values of 625 *μ*g/mL and 1500 *μ*g/mL, respectively.

## 4. Discussion

Plants are well-known to have beneficial effects on human health. Their extraordinary wealth of biologically active compounds makes them indispensable for human life and allow the prevention of many civilization diseases. One plant species that contains many valuable biologically active compounds is *Menyanthes trifoliata*. Extracts and infusions of this plant are used as anti-inflammatory, diuretic, or cleansing agents, becoming an important element of phytomedicine [20].

This work is the first to demonstrate the protective properties of *M. trifoliata* extracts derived from aerial parts and roots of plants cultivated *in vitro*. It examines the anti-inflammatory and antioxidant properties of *M. trifoliata* extracts (MtRV and MtAPV extracts) in human umbilical vein endothelial cells (HUVECs) which had previously been treated with LPS or H_2_O_2_. Inflammation is a very complex physiological phenomenon developing in the tissue under the influence of different stimuli and is mediated by a wide range of specific components. It involves interactions between signal molecules produced by leukocytes, macrophages, and mast cells, as well as by the activation of complements [21–23]. A range of studies describe the anti-inflammatory properties of plant extracts against a wide spectrum of normal and cancer cells [24–26].

One of the aims of the study was to investigate the anti-inflammatory properties of *M. trifoliata* extracts on HUVECs following stimulation by lipopolysaccharide (LPS) for 24 hours. LPS is the main component of the outer membrane of Gram-negative bacteria. It activates mononuclear phagocytes and other cell types by toll-like receptor 4 to promote the secretion of inflammatory mediators including TNF-*α*, IL-6, and IL-1*β* [27]. It was found that the aerial part (MtAPV) and root (MtRV) extracts of *in vitro*-derived *M. trifoliata* exhibit an anti-inflammatory effect by decreasing the expression of selected genes encoding inflammation-associated cytokines (IL-1*α*, IL-1*β*, IL-6, TNF-*α*, and IFN-*γ*).

Many reports indicate that *M. trifoliata* is a source of phenolic compounds such as coumarins, flavonols, or iridoids [11]. Our previous study also showed the presence of various phenolic compounds in the extracts of *in vitro*- and *in vivo*-derived *M. trifoliata* plants, with the content of ellagic acid (299-518 *μ*g/g dry weight), chlorogenic acid (129-258 *μ*g/g dry weight), and rutin (82-256 *μ*g/g dry weight) varying according to the plant part and growth conditions. Betulinic acid, a pentacyclic triterpene, was found at its highest concentrations in the roots of plants derived from *in vitro* cultures. The anti-inflammatory and antioxidant activity of this plant compound is also well documented in literature [28–30].

Our present findings indicated that the tested *M. trifoliata* extracts bestow a DNA-protective effect on the tested cell line. We speculate that betulinic acid and the identified phenolic acids (syringic acid, sinapinic acid, ellagic acid, and chlorogenic acid) may be responsible for this positive effect, especially as betulinic acid and sinapinic acid have been found to suppress inflammatory cytokines such as IL-6 or TNF-*α* [31, 32]. Betulinic acid (lupane-type triterpene) exhibits a protective effect on mice exposed to lethal doses of LPS and causes a reduction of LPS-induced TNF-*α* production. In addition, betulinic acid is also known as an inhibitor of IFN-*γ* production [33]. Kim et al. report the compound to be an anti-inflammatory agent acting via inhibition of the nuclear factor-*κ*B (NF-*κ*B) pathway. Similarly, suppression of tumor necrosis factor-*α* (TNF-*α*), interleukin-6 (IL-6), and interleukin-1*β* (IL-1*β*) was demonstrated in LPS-activated RAW 264.7 macrophages after betulinic acid treatment [34]. Similar properties have also been described for sinapinic acid [35–37], ellagic acid [38], or chlorogenic acid [39].

The present study also analyzes the influence of the two *M. trifoliata* extracts on oxidative stress-related gene expression. Changes in the expression of genes encoding heme oxygenase 1 (HO-1), quinone dehydrogenase 1 (NQO1), nuclear factor (erythroid-derived-2)-like-2 (NRF2), Kelch-like ECH-associated protein 1 (kEAP1), or glutamate-cysteine ligase catalytic subunit (GCLC) cast light on the protective role played by the components of the tested extract in H_2_O_2_-stimulated cells. The MtRV and MtAPV extracts, administered at 1 mg/mL, were found to influence the expression of *HO-1*, *NRF2*, *kEAP1*, *NQO1*, and *GCLC* genes in H_2_O_2_-stimulated HUVECs, with the MtRV extract demonstrating better protective properties than the MtAPV.

Previous studies provide a wealth of information describing the cytoprotective effect of plant phenolic compounds on HUVECs [40–42]. Some affect the expression of oxidative stress-related genes; for example, those contained in the green tea extract increase the mRNA level of heme oxygenase-1 (HO-1) in human aortic endothelial cells [43], while the polyphenolic antioxidant fraction from *Nymphaea nouchali* leaves increases HO-1 and Nrf2 level in RAW 264.7 cells [44]. Recently, special attention has been paid to the close relationship between inflammation and oxidative stress. The appearance of an excessive amount of free radicals reacts with cell membrane fatty acids, proteins, or DNA, leading to mutation and consequently to the development of many diseases. In this context, searching for natural components that protect cells from adverse changes is extremely desirable.

The present study also tested cytoplasmic and mitochondrial ROS levels in H_2_O_2_-stimulated HUVECs after treatment with MtRV and MtAPV extracts. Our results, obtained with dichloro-dihydro-fluorescein diacetate and red mitochondrial superoxide indicator (MitoSOX), found the plant extracts to exert a protective effect by decreasing cytoplasmic and mitochondrial ROS levels in cells. The MtRV extract demonstrated a stronger antioxidant effect than the MtAPV extract. It is known that ROS are produced in epithelial cells by many enzymes, including nicotinamide adenine dinucleotide phosphate oxidase (NOX), nitric oxide synthase (NOS), or xanthine oxidase (XOD), as well as the respiratory chain complex [45]. ROS are extremely important elements that play a crucial role in the initiation of inflammatory processes. Elevated levels of ROS produced by polymorph nuclear neutrophils (PMN) at the site of inflammation can lead to epithelial cell dysfunction and consequent tissue damage [46]. A considerable body of evidence indicates that the excessive cellular production of ROS may be closely related to the induction of inflammation and consequently affect the development of diseases such as Alzheimer's, Parkinson's, or cancer [47]. Our findings confirm that *M. trifoliata* extracts exhibit protective antioxidant properties, probably due to the presence of identified phenolic compounds and pentacyclic triterpenes, such as betulinic acid, which is consistent with literature data [48]. It should also be noted that the MtRV extract appears to offer the strongest cytoprotective effects.

Nuclear and mitochondrial DNA damage was also quantified by SLR-qRT-PCR amplification of DNA from HUVECs exposed to plant extracts followed by 50 *μ*M H_2_O_2_. Both extracts were found to have a protective effect, resulting in a decrease in the lesion rate for all the tested nuclear and mitochondrial DNA regions after incubation with MtRV and MtAPV extracts compared to cells incubated only with 50 *μ*M H_2_O_2_. Although mitochondrial and nuclear DNA damage, and hence the protective effects of the tested extracts, can be detected by various methods such as Southern blot analysis or comet assay, the present study used a semilong run RT-PCR-based assay. Also, the MtRV extract demonstrated a stronger protective potential against ROS-derived DNA damage than the MtAPV extract.

Plant phenolic compounds are known to bestow a protective effect on DNA against various physical or chemical factors [49–51]. A similar study investigating the protective effect of *Salvia officinalis* and *Thymus vulgaris* extracts on the DNA of HepG2 cells was performed by Kozics et al. [52]. Their results indicate that H_2_O_2_-induced DNA damage was significantly reduced in cells pretreated with both the tested plant extracts, which were also found to contain phenolic compounds and betulinic acid. Another study examined the effect of a purified *Eugenia jambolan* leaf and fruit extract on HepG2 cells; the extracts were found to be rich sources of many phenolic compounds and betulinic acid and to exhibit antigenotoxic and antimutagenic effects [53].

Other studies have shown that phenolic compounds, including phenolic acids, can reduce the level of oxidative DNA damage in a variety of cell types [54, 55]. However, the present study is first to report the ability of the *in vitro*-derived *M. trifoliata* plant extract to protect cells against DNA damage. As noted in previous studies, our results suggest that the protective effect demonstrated by the examined extracts is related to its phenolic compound content, including betulinic acid, and the synergy between extract components.

Flow cytometric analysis showed that *M. trifoliata* extracts had a protective effect on the tested cells. HUVECs treated with H_2_O_2_ generated a higher level of *γ*H2A.X- and cleaved PARP1-positive cells than untreated cells. On the other hand, 24-hour incubation with the studied MtRV and MtAPV extracts exhibited a protective effect against pretreatment with H_2_O_2_ by reducing the number of *γ*H2A.X- and cleaved PARP1-positive cells. Poly (ADP-ribose) polymerase 1 (PARP1) is responsible for the ADP-ribosylation process (PARylation), which is a common posttranslational modification of DNA damage. This modification regulates many other biological processes, including the reorganization of chromatin or DNA damage response (DDR). In addition, PARP is a substrate for caspases and is cleaved during apoptosis into 86 and 24 kDa fragments [56, 57]. For this reason, PARP1 acts as a good DNA damage sensor [58]. Similarly, the H2A.X protein is a key factor in the repair of damaged DNA and can be phosphorylated on the 139th serine residue in the presence of DNA damage [59]. Phosphorylated protein causes a conformational change in the DNA-H2A.X complex, which allows recruitment of proteins needed to repair DNA injury [60].

Our results clearly show that *M. trifoliata* extracts from plants derived from *in vitro* cultures play an important role in DNA protection of the tested cells. This is consistent with other literature data showing that polyphenol-rich natural products also inhibit the increases in cleaved PARP level [61, 62]. Similarly, the level of phosphorylated H2A.X, another determinant of DNA damage, has previously been found to be reduced under the influence of the black tea or *Camptosorus sibiricus* extract [63] in other cells. It is most likely that the phenolic compounds and pentacyclic triterpene present in the MtRV and MtAPV extracts may be responsible for the biological effect observed in the present study.

The study also examines the antibacterial and antifungal properties of *M. trifoliata* extracts. The tested MtRV and MtAPV extracts demonstrated stronger antibacterial than antifungal action. It was also shown that the MtRV extract possessed a stronger effect against the tested microorganisms than MtAPV. The best bacterial growth inhibitory effects were obtained against *Pseudomonas aeruginosa* (ATCC 27853) and *Enterococcus faecalis* (ATCC29212) for the MtRV extract (MIC = 150 *μ*g/mL). Similar effects were demonstrated for the MtAPV extract against all the tested bacteria (MIC = 250 *μ*g/mL). Also, better fungal growth inhibition was exhibited by the MtRV extract than the MtAPV extract against *Candida albicans* (ATCC 10231) and *Saccharomyces cerevisiae* (ATCC 2601), where the MIC for *Candida albicans* was 625 *μ*g/mL. Many plant species naturally produce compounds with antimicrobial or antifungal properties [64–66]. Ivanišová et al. [67] examined the antimicrobial activity of *M. trifoliata* leaf extracts, rich sources of polyphenols and flavonoids, and extracts from other plants against three Gram-negative bacteria and two Gram-positive species. The *M. trifoliata* extract demonstrated the strongest antibacterial activity against *Escherichia coli*, *Salmonella enterica* subsp. *enterica*, *Bacillus thuringiensis*, *Staphylococcus aureus* subsp. *aureus*, and *Pseudomonas aeruginosa*, which corresponds to our results. This study found *M. trifoliata* to be the richest source of polyphenols, flavonoids, and phenolic acids among all the analyzed wild plants in the study. The antibacterial and antifungal properties of *M. trifoliata* extracts have also been investigated by Paudel et al. [68] by testing their influence on pathogenic microorganisms such as *S. aureus*, *E. coli*, and *C. albicans*. In this case, the plant extracts were active only against *S. aureus*. The authors attribute these differences in antimicrobial activity to variations in antimicrobial metabolites among the tested samples.

The search for new antibacterial compounds has recently become an extremely important issue due to emerging resistance of pathogens to traditional medicines; in this sense, plant-derived compounds can play a potentially important role. They often have specific chemical structures that can inhibit bacterial growth through new mechanisms of action [69]; however, this group of secondary metabolites may also be of great importance in the development of new compounds against human pathogens. Based on data obtained in previous studies [70–72], it is likely that betulinic acid may be chiefly responsible for the antibacterial and antifungal effects of the tested extracts [70, 73]. Additionally, we suspect that phenolic acids may have a similar effect and show synergistic effects with other components, particularly since the MtRV extract showed the best antimicrobial properties. Generally, the antibacterial and antifungal mechanism of action of many phenolic compounds is not yet fully understood [69]. Some studies indicate that they interact with a range of targets such as DNA, DNA gyrase [74], protein kinases [75], and helicase, among others [69]. Attempts should be made to identify new antimicrobial components, especially as increasing numbers of multidrug-resistant strains are appearing in the environment [76].

## 5. Conclusion

The results of our study demonstrate for the first time that MtRV and MtAPV extracts from *M. trifoliata* plants derived from *in vitro* cultures showed antioxidant and anti-inflammatory effects on human umbilical vein endothelial cells (HUVECs) stimulated by H_2_O_2_ or LPS, and of the two, the MtRV extract demonstrated the strongest activities. Additionally, the protective effect was observed which was associated with a decrease in DNA damage, reductions in cytoplasmic and mitochondrial ROS levels, and reduced *γ*H2A.X- and cleaved PARP1-positive HUVEC numbers. Furthermore, the tested extracts demonstrated moderate antimicrobial activities against various pathogenic bacterial and fungal strains. Therefore, this plant may play a potential role in the therapy and prevention of many civilization diseases.

## Figures and Tables

**Table 1:** Chosen secondary metabolites of aerial parts (MtAPV) and roots (MtRV) of *Menyanthes trifoliata* plants grown *in vitro* [8].

Compounds	MtRV	MtAPV
	*μ*g/g dry weight	
Betulinic acid	5437.15 ± 141.33	395.31 ± 14.50
Chlorogenic acid	177.34 ± 9.57	257.9 ± 14.70
Ellagic acid	518.11 ± 26.46	450.65 ± 14.22
Rutin	256.20 ± 3.24	152.99 ± 6.24
Sinapinic acid	146.53 ± 7.03	71.16 ± 3.34
Syringic acid	113.80 ± 0.80	n.d.

**Table 2:** Antimicrobial activity of MtRV and MtAPV extracts of *M. trifoliata*. Van: vancomycin; NOR: norfloxacin; ANF: amphotericin B. Data represent the median values in triplicate.

Plant material	*Staphylococcus aureus*		*Pseudomonas aeruginosa*		*Escherichia coli*		*Enterococcus faecalis*		*Saccharomyces cerevisiae*		*Candida albicans*	
	MIC (*μ*g/mL)	MBC	MIC (*μ*g/mL)	MBC	MIC (*μ*g/mL)	MBC	MIC (*μ*g/mL)	MBC	MIC (*μ*g/mL)	MFC	MIC (*μ*g/mL)	MFC
Mt RV extract	**225**	>**500**	**150**	>**500**	**250**	>**500**	**150**	>**500**	**725**	**625**	**625**	**1500**
Mt APV extract	**250**	>**500**	**250**	>**500**	**250**	>**500**	**250**	>**500**	**725**	**725**	**925**	**2500**
Positive control	**7.82** VAN	>**500**	<**0.48** NOR	>**500**	**0.98** NOR	>**500**	**1.95** VAN	>**500**	<**0.48** ANF	>**5000**	<**0.48** ANF	>**5000**

## Data Availability

All data are available at the request of the reviewer or other interested persons.
